# Human dignity of patients with cardiovascular disease admitted to hospitals of Kerman, Iran, in 2015

**Published:** 2016-07-16

**Authors:** Roghayeh Mehdipour-Rabori, Abbas Abbaszadeh, Fariba Borhani

**Affiliations:** 1 Assistant Professor, Department of Medical-Surgical Nursing, Razi School of Nursing and Midwifery, Kerman University of Medical Sciences, Kerman, Iran;; 2 Professor, Department of Medical-Surgical Nursing, School of Nursing and Midwifery, Shahid Beheshti University of Medical Sciences AND Academy of Medical Sciences, Tehran, Iran;; 3 Associate Professor, Medical Ethics and Law Research Center, Shahid Beheshti University of Medical Sciences, Tehran, Iran.

**Keywords:** *Heart disease*, *Human dignity*, *Patient rights*, *Iran*

## Abstract

The human dignity of patients with cardiovascular disease (CVD) is an important issue, because of patients’ dependence upon caregivers, and because it impacts all aspects of their quality of life (QOL). Therefore, understanding and improving the status of dignity among these patients is of great importance. This study aimed to determine the status of dignity in patients with CVD admitted to cardiac intensive care units (CICUs) in Iran.

This cross-sectional descriptive study was performed in 2015 on 200 patients admitted to the CICUs of hospitals affiliated to Kerman University of Medical Sciences, Iran. The participants were selected using random sampling method. Patients’ understanding of dignity was assessed through the reliable and valid Persian version of the Patient Dignity Inventory (PDI). Patients who were able to read and write or speak Persian and were conscious were included in the study. Data were analyzed using descriptive statistics tests, independent t-test, and one-way ANOVA in SPSS software.

The mean age of the study participants was 59.0 ± 17.0. The mean score of human dignity was 3.60 ± 1.39. The mean scores of the factors of loss of independence, emotional distress and uncertainty, changes in ability and mental image, and the loss of human dignity were 3.94 ± 1.06, 3.63 ± 1.37, 3.57 ± 1.20, and 3.30 ± 2.08, respectively. A significant statistical correlation was observed between human dignity and the demographic characteristics of gender and frequency of hospitalizations in a CICU and a significant difference between those who lived alone and those who lived with family was observed (*P* < 0.05).

Patients hospitalized in CICUs experience numerous problems associated with human dignity in each of its four dimensions. It is recommended that a study be conducted to investigate the relationship between the human dignity of patients with CVD and their QOL, anxiety, and depression.

## Introduction

The term dignity is derived from the Latin words *dignitus,* meaning competency, and *dignus,* meaning derived value (1). Although the term ‘human dignity’ has been used in numerous studies, its meaning is not clear (2). A simple definition of human dignity is the intrinsic value of an individual due to being human. A sense of this value is formed in the individual through relationships with others (3). Human dignity has always been considered as important in all countries and religions and it is the foundation of human rights (4). Illness, disability, need, reduced power and authority, lack of privacy, and being treated and hospitalized can affect human dignity (3). The restriction of human dignity can affect the body, spirit, morality, and spirituality of patients and expose them to stress and discomfort (3). 

Different studies have shown that patients and nurses, depending on the culture of their country and region, have different definitions for and expectations regarding their dignity (5). Manookian et al. defined the concept of patient dignity from the perspective of nurses as respect for the dignity of human nature, provision of professional patient-centered care, and respect for the rights of patients’ relatives (6). Baillie also referred to patient dignity as consisting of feelings (feeling relaxed, in control, and respected), physical performance, and behavior. The environment, patient behavior, and factors related to healthcare personnel impact patient dignity (7). In a review study, Shahriari et al. reported that human dignity is considered a trivial matter and accepted as part of the patient rights (8). Different studies have shown that a lack of understanding of the concept of patient dignity may cause the treatment process to become difficult or may prevent patients from returning to treatment centers (9). 

Studies indicate that, in recent years, nurses have become interested in issues such as the dignity of children, rehabilitation, patients in need of long-term care, and end-of-life patients (5, 6, 10). However, a limited number of studies have been conducted on dignity and its importance in patients who have been hospitalized in cardiac intensive care units (CICUs). 

Cardiovascular disease (CVD) is a major, universal public health problem. In the United States, CVD is an important cause of death. According to the U.S. National Vital Statistics Report, 315,706 men and 315,930 women died of CVD in 2006, which accounted for about 26% of all deaths (11). It is estimated that in developing countries, because of changes in eating habits and the promotion of a sedentary lifestyle, CVD has become one of the most common causes of death (12). In Iran, CVD is the most common cause of death (13). This disease affects patients, families, and society, causes the social isolation of patients, reduces quality of life (QOL), and causes dissatisfaction (14).

CVD results in patients’ dependence on their caregivers (nurses and family). This situation can affect human dignity and lead to patients’ loss of dignity (4). 

Studies have shown that patients who suffer from chronic diseases like kidney disease, multiple sclerosis (MS), high blood pressure, diabetes, stroke, and psychiatric diseases also suffer from CVD (15-17).

 Studies have indicated that respecting patients dignity in CICUs increases patient’s satisfaction and certainty of care, reduces length of hospitalization, and increases patient’s mental health (18). Valian et al. also indicated that showing respect for patients increases patient satisfaction (19). Onal and Civaner found that one expectation of patients regarding preservation of their rights was respecting human dignity (20). 

It has been recognized that patients are not well respected in hospitals. In one study, Baillie has indicated that patients in London are subjected to a reduction in dignity (21). In Iran, Ebrahimi et al. conducted a literature review and declared that patient dignity is not well supported and that medical and nursing staff do not have a correct understanding of patient dignity (22). Respect for human dignity is the basis of nursing care, and one of the responsibilities of nurses is providing mental health care for patients. The different aspects of human dignity and the difficulties and discomforts that patients experience in this regard, however, are unknown. Little research has been conducted on the dignity of patients in CICUs. 

Because of the importance of patient dignity and lack of studies in this field which can indicate the current status and requirements of patients, this study was conducted at the aim of assessing the status of patients dignity in CICUs. It is hoped that by identifying the status of patients dignity in CICUs, nurses and other healthcare providers may try to resolve problems related to human dignity and promote patients dignity.

## Method

This cross-sectional descriptive study was conducted in Kerman, Iran, in 2015. It investigated the status of human dignity in patients with cardiovascular disease (CVD). The study population consisted of cardiac patients hospitalized in CICUs in hospitals affiliated to Kerman University of Medical Sciences, Iran. 

The researchers used a two-part questionnaire to collect data. The first part was a demographic questionnaire consisting of items on gender, marital status, education, occupational statues, and frequency of hospitalization (Table 1). The second part was the Patient Dignity Inventory (PDI). This tool was designed in 2008 by Chochinov et al. and is used to measure human dignity and various sources of distress and discomfort associated with patient dignity (23). In 2015, the validity of the PDI in Iranian patients with CVD was approved by Abbaszadeh et al. (24). This questionnaire includes 4 dimensions; loss of human dignity (11 items), emotional distress and uncertainty (8 items), changes in ability and mental image (4 items), and loss of independence (2 items) (24). Table 1 provides more details about each dimension. Each item on this questionnaire is scored between 1 and 5 in terms of the patient’s response, with a score of 1 indicating lack of any problems and 5 indicating presence of severe problems. The higher the score is, the greater is the problem associated with patient dignity. Therefore, a score of 3 or higher indicates a serious problem. 

This standard 25-item tool has been previously investigated on a large number of patients. Its internal correlation coefficient was 0.93, and its reliability in retest was 0.85 (25).

In order to determine the face validity of the questionnaire, it was given to 20 patients with CVD with different educational levels. As a result, a face validity of 0.95 was obtained. Cronbach’s alpha was used to determine the reliability of PDI (α= 0.87). 

To collect data, the Persian version of the questionnaire was distributed among patients with CVD hospitalized in CICUs. Those patients who were able to read and write completed the questionnaires themselves, and patients who were illiterate completed the questionnaires through an interview with someone tending the patient. Conscious patients whose health condition did not allow them to answer the questionnaire completed it when stabilized.

To determine the sample size, the researchers first conducted a pilot study in which 20 randomly-selected patients hospitalized in a CICU completed the PDI. After calculating mean and standard deviations and taking into account a 95% confidence interval, an accuracy of 0.05, and S^2^ of 1296, sample size was estimated as 200 individuals. Thus, 200 patients hospitalized in the CICUs of educational hospitals of Kerman were selected using random sampling method in 6 to 7 months. Patients who were hospitalized in CICU, conscious, able to read and write or speak Persian, and able and willing to participate in the study were included. Patients who were hospitalized for the first time in the intensive care unit (ICU) were excluded from the study.

The permission to conduct the study was obtained from the Ethics Committee of the Medical Sciences Research Center (Ethical Code: K93.317). The researchers explained the study to the patients and signed informed consent forms were obtained from all patients participating in the study before the completion of questionnaires. They were assured that their information would be kept confidential. Participation or non-participation in the study had no effect on the process of care. The collected data were analyzed using SPSS software (version 13, SPSS Inc., Chicago, IL, USA). Descriptive analysis tests (mean, SD, and frequency) and the independent t-test were used to compare human dignity in terms of gender, marital status, number of hospitalizations, and lifestyle. One-way ANOVA was used to compare human dignity in terms of education level and occupation. All *P* values of less than 0.05 were considered significant.

## Results

Data analysis indicated that of the 200 patients, 117 patients were men and 83 were women. The average age of the participants was 59.0 ± 17.0. Other demographic data are presented in table 1. The mean score of human dignity was 3.60±1.39, and data analysis indicated that patients mentioned problems in all four dimensions. The mean scores of categories of loss of independence, emotional distress and uncertainty, changes in ability and mental image, and loss of human dignity were 3.94 ± 1.06, 3.63 ± 1.37, 3.57 ± 1.20, and 3.30 ± 2.08, respectively.

The items of uncertainty about the disease and treatment process (4.83 ± 0.17), the feeling of being a burden to others (4.62 ± 0.38), having physically distressing symptoms (4.59 ± 0.41), the feeling that sickness and care have caused an invasion of privacy (4.52 ± 0.46), anxiety (4.36 ± 0.64), the feeling of emotional inability to cope with the challenges of the disease (4.16 ± 0.84), feeling one has changed in appearance (4.04 ± 0.17), and inability to perform daily living tasks (4.01 ± 0.99) had, respectively, the highest to lowest average scores. Only two items had average scores of less than 2; the feeling that one is not the same person as before (1.89 ± 3.11) and the feeling that one has not made a meaningful contribution (1.38 ± 3.62) (Table 1).

**Table 1:** The mean scores of patient dignity

**Dimensions and Items**	**Mean ± SD**
**Loss of human dignity**	
Not feeling worthwhile or valued	3.27 ± 2.93
Not able to carry out important roles	3.16 ± 2.44
Feeling life no longer has meaning or purpose	3.14 ± 1.16
Feeling that one has not made meaningful contributions	1.38 ± 3.62
Feeling one has unfinished business	2.84 ± 2.16
Concerns regarding spiritual life	3.16 ± 2.84
Not feeling in control	3.91 ± 1.09
Reduced privacy	4.52 ± 0.46
Not feeling supported by friends	2.12 ± 2.88
Not feeling supported by healthcare providers	2.83 ± 2.17
Not being treated with respect	3.89 ± 1.11
**Emotional distress and uncertainty**	
Feeling depressed	2.90 ± 2.10
Feeling anxious	4.36 ± 0.64
Feeling uncertain	4.83 ± 0.17
Worried about future	3.62 ± 1.38
Not being able to think clearly	2.04 ± 2.96
Feeling that one is a burden to others	4.63 ± 0.38
Not being able to overcome the challenges of the illness	4.16 ± 0.84
Not being able to accept the way things are	2.50 ± 2.49
**Changes in ability and mental image**	
Physically distressing symptoms	4.59 ± 1.41
Feeling one has changed in appearance	4.04 ± 0.17
Not being able to continue usual routines	3.76 ± 0.13
Feeling one is not the same person as before	1.89 ± 3.11
**Loss of independence**	
Not able to perform daily living tasks	4.01 ± 0.99
Not able to attend to bodily functions	3.87 ± 1.13

Table 2 compares mean human dignity scores in terms of the demographic characteristics of patients in the current study. As can be seen, there is a positive statistical relationship between the mean score of human dignity and the demographic characteristics of gender, frequency of hospitalizations in the CICU, and lifestyle. Mean human dignity score was higher in women than men (*P* = 0.001). The mean score of human dignity was also higher in those who had been hospitalized more than 3 times than in those who had been hospitalized less than 3 times (*P* = 0.010). Moreover, the mean score of human dignity was higher in patients who lived alone than in those who lived with their family (*P* = 0.001).

**Table 2:** Demographic data and human dignity scores of the participants

**Demographic variables**		**Total sample = 200 n (%)**	**Mean score of patient dignity**	***P***
Gender	Men	117 (58.5)	2.80 ± 0.95	0.001**
	Women	83 (41.5)	4.41 ± 1.84	
Marital status	Yes	135 (67.5)	3.39 ± 1.37	0.200**
	No	65 (32.5)	3.81 ± 1.41	
Education	None or primary education	135 (67.5)	3.68 ± 1.32	0.100*
	High school	41 (20.5)	3.52 ± 1.13	
	College	19 (9.5)	3.73 ± 1.46	
	Postgraduate	5 (2.5)	3.47 ± 1.65	
Occupational status	Employed	61 (30.5)	3.44 ± 2.49	0.500*
	Unemployed	42 (21)	3.40 ± 1.01	
	Retired	24 (12)	3.75 ± 1.31	
	Housewife	73 (36.5)	3.81 ± 0.75	
Frequency of hospitalizations	Less than 3 times	137 (67.5)	3.10 ± 1.50	0.010**
	More than 3 times	63 (31.5)	4.11 ± 1.29	
Lifestyle	Live alone	20 (10)	4.47 ± 2.40	0.001**
	Live with family	180 (90)	2.73 ± 0.38	

* Note: ANOVA,

** T-test

## Discussion

The present study indicated that the level of human dignity felt by patients hospitalized in CICUs is low. This finding is similar to the findings of other researches, including studies by Hall et al. and Vehling and Mehnert (26, 27). However, only a few studies had been conducted on the dignity of patients with CVD. Patients with CVD feel that their human dignity is threatened because of their dependence on family, the chronic nature of their disease, and repeated hospitalizations (28). Thus, one of the problems of patients during hospitalization is the loss of dignity (29).

 The present study indicates that patients with CVD have problems in all 4 dimensions of human dignity. Regarding changes in ability and mental image, the results of earlier studies have indicated that when a disease is severe, the problems associated with patient dignity, ability, and mental image are also severe. This is due to their dependence upon families and caregivers. Physical pain also causes problems for them, and patients with CVD are not excluded from this trend (30, 31).

Patients in the current study reported experiencing problems related to human dignity in the dimension of emotional distress and uncertainty. In a study conducted by Biragh et al., 72% of patients with CVD reported symptoms of depression and 90% of the patients reported anxiety symptoms (31). Denollet et al. indicated that a decrease in anxiety and psychological distress improves patients with CVD (32). This finding suggests that patients with CVD experience mental health problems due to their disease. The nature of CVD is such that it affects various aspects of an individual’s life, such as comfort, lifestyle, income, and occupation. Nurses should consider this and attempt to rehabilitate patients with CVD in a way that promotes human dignity.

Patients experienced problems related to human dignity after loss of independence. Patients, who are able to make reasonable and rational choices, are competent in having authority, and are able to consciously make decisions based on their knowledge and the information provided them and participate in the mutual cooperation related to their healthcare (33). Most patients with CVD are capable of making rational and informed decisions. If they have a problem related to dignity after losing authority, it is significant. Nurses should pay sufficient attention to this matter and should involve these patients in their treatment process by providing them with information.

The findings of this study indicate that there is a significant relationship between gender and emotional problems related to human dignity; women feel more problems associated with human dignity than men. Also Vehling and Mehnert, in their study, concluded that women experience more problems associated with human dignity than men (27). In a study conducted by Hall et al., however, no relationship was found between gender and problems associated with human dignity (26). This difference may be due to cultural differences between Iran and England. Gender differences between men and women in the Iranian family and men’s stronger support system are perhaps the causative factors of this difference.

The current study also indicated that there is a significant relationship between the number of hospitalizations and problems related to patient dignity. Number of hospitalizations is affected by the severity and duration of the disease. Studies have shown that there is a relationship between type of illness and dignity, and this is why the longer the duration of the disease, the more patient dignity is affected and reduced (34,35). 

This study indicated that there is a statistically significant correlation between living alone and problems associated with human dignity (36). Those living alone reported more problems related to human dignity than those living with family. This, too, could be affected by the culture of a country where one tends to have a strong family support system during illness. Human dignity is affected by distress; living alone is a cause of distress and is stressful for Iranian individuals. A subcategory of dignity is social dignity, which is formed through relationships with others (37). The lower the rate of social interactions, the more social dignity is reduced.

In the study conducted by Sautier et al. in Germany on the dignity of patients with cancer, no significant difference was found between demographic characteristics and the dignity of patients (25). This difference in findings is probably due to differences of the participants studied by Sautier et al. with those in the present study. All patients studied by Sautier et al. had an advanced-stage cancer and numerous experiences of hospitalization. The difference in the disease of the participants also created differences between the results of that and the current study.

All items of the questionnaire used in the current study are valuable and important in patient dignity. A score of lower than 3 does not mean the item is less significant; each item is of great importance, because each one alone has a profound impact on dignity. Considering that nurses spend most of their time with patients in hospitals and that patients with CVD are dependent upon their caregivers, nurses can take steps to respect patient dignity and educate patients’ families to preserve and promote patient dignity. Through identifying the problems associated with patient dignity, attempts can be made to overcome these problems and steps can be taken to raise the level of human dignity in patients with CVD hospitalized in CICUs.

## Conclusion

The present study showed that patients with CVD have difficulties in maintaining their dignity. Hence, nurses and other health providers should provide assistance to these patients. 

This study had some limitations. One limitation was that only conscious patients were enrolled. Moreover, the study was conducted in teaching hospitals (public), and differences may exist between patients in CICUs of teaching hospitals (public) and those of non-teaching hospitals (private). It is recommended that future studies compare levels of human dignity between patients hospitalized in public and private hospitals.
